# Illicitly Manufactured Fentanyl Entering the United States

**DOI:** 10.7759/cureus.17496

**Published:** 2021-08-27

**Authors:** Joseph Pergolizzi, Peter Magnusson, Jo Ann K LeQuang, Frank Breve

**Affiliations:** 1 Cardiology, Native Cardio Inc., Naples, USA; 2 Cardiology, Center of Research and Development Region Gävleborg/Uppsala University, Gävle, SWE; 3 Medicine, Cardiology Research Unit, Karolinska Institute, Stockholm, SWE; 4 Pain Management, NEMA Research Inc., Naples, USA; 5 Department of Pharmacy, Temple University, Philadelphia, USA

**Keywords:** opioid epidemic, fentanyl, immigration, drug smuggling, narco trafficking, fentanyl analogs, illicit fentanyl, mexico, china

## Abstract

The 'third wave' of the ongoing opioid overdose crisis in the United States (US) is driven in large measure by illicitly manufactured fentanyl (IMF), a highly potent synthetic opioid or an analog developed in clandestine laboratories primarily in China and Mexico. It is smuggled into this country either as IMF or as precursors. The southern border of the US is a frequent point of entry for smuggled IMF and the amounts are increasing year over year. IMF is also sometimes mixed in with other substances to produce counterfeit drugs and dealers as well as end-users may not be aware of IMF in their products. IMF is inexpensive to produce and when mixed with filler materials can be used to cut heroin, vastly expanding profitability. It is an attractive product for smuggling as very tiny amounts can be extremely potent and highly profitable. Drug trafficking over the border also involves the tandem epidemic of money laundering as drugs enter the country and cash payments exit. While drug smuggling in and out of the US (and other nations) has been going on for decades, the patterns are changing. Highly potent and potentially lethal IMF is a dangerous new addition to the illicit drug landscape and one with disastrous consequences.

## Introduction and background

**Figure 1:**
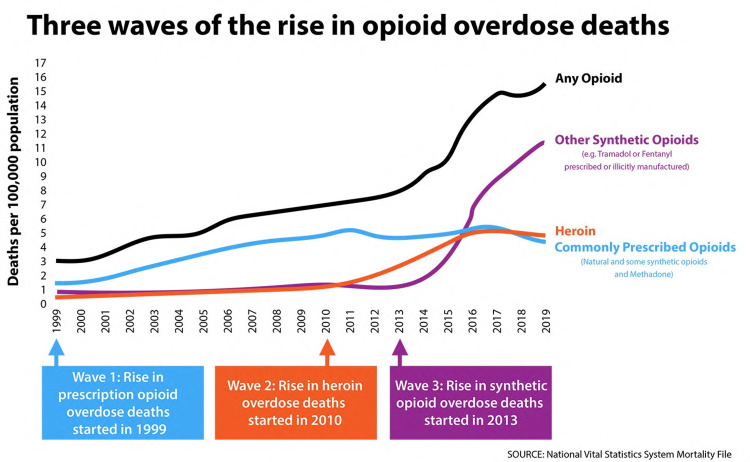
The opioid epidemic in the United States has been defined as having three distinct waves.

In 2019, 70,630 people died of a drug overdose in the United States (US), an increase of 4% over the preceding year; from 20.7 to 2l.6 per 100,000. Most drug overdose deaths (70.6%) in the US involve an opioid; synthetic opioids, such as illicitly manufactured fentanyl (IMF), are thought to be driving the increased mortality. It is sobering to note that no state in the entire US saw a decrease in overdose mortality from 2018 to 2019 [1]. However, from 2018 to 2019, the rate of heroin-involved overdose deaths dropped by 6% [2]. A geographical shift has also occurred with significant increases in synthetic opioid overdose deaths reported in eight states west of the Mississippi (Arizona, California, Colorado, Minnesota, Missouri, Oregon, Texas, and Washington), whereas earlier most deaths associated with synthetic opioids were concentrated east of the Mississippi [1,3]. This new increase in opioid mortality has been called the 'third wave' of opioid overdose deaths and commenced around 2013 with the influx of synthetic opioids such as IMF [4]. See Figure 1.

The aim of this narrative review was to explore the role of IMF in the current increase of opioid-related deaths, especially in light of activity at the Southern border into the US.

## Review

**Figure 2:**
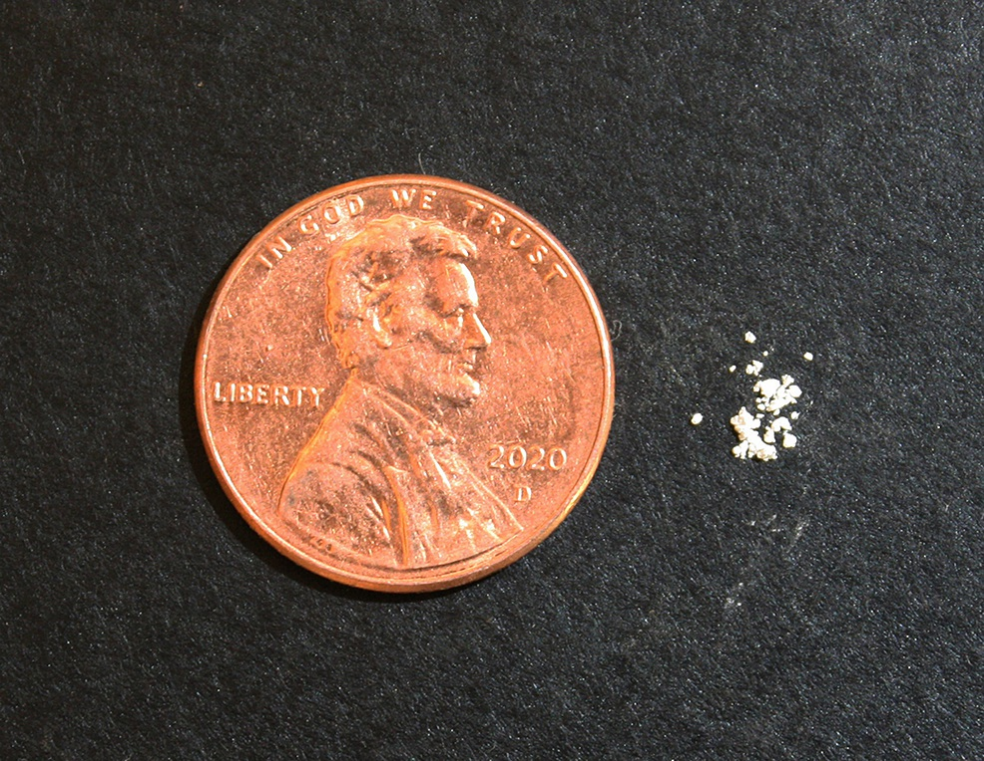
About 2 mg of a harmless powder representing the amount that would be a lethal dose of intravenous illicit fentanyl. Potency is defined as the measure of a drug’s effect relative to dose and is assessed in terms of intravenous use of the drug. Fentanyl is 50 to 100 times more potent than morphine. While fentanyl may be absorbed cutaneously, a small amount of fentanyl in brief contact with intact skin is usually insufficient to cause intoxication. Inhalation of fentanyl or fentanyl in contact with mucus membranes is potentially more dangerous [6].

Fentanyl is a synthetic opioid that can be 50 to 100 times as potent as morphine; prescription fentanyl products are available for use in surgery and pain control for severe cancer pain, but the drug and its analogs are comparatively easy and inexpensive to manufacture in clandestine laboratories [5]. IMF is often mixed with filler materials (such as lactose, mannitol, and sugars) and then cut into heroin to increase product volume and provide street dealers better economic returns. Filler with IMF can also be pressed using pill presses into counterfeit 'prescription' oral opioids, such as fake oxycodone. The admixtures are exceedingly dangerous, as the potency of illicit fentanyl is so great that even minuscule quantities can be fatal [5]. See Figure 2. Furthermore, IMF is manufactured in clandestine laboratories and is of variable strength, purity, and quality.

The first seizure of illicit fentanyl at the US border with Mexico was reported in April 2016 when the Drug Enforcement Administration (DEA) seized hundreds of counterfeit oxycodone tablets that contained IMF. The drugs were seized at the Otay Mesa Port of Entry in the possession of a pedestrian, who admitted he was smuggling the drugs into the US [7]. The DEA has declared that 'fentanyl is the most prevalent and the most significant synthetic opioid threat to the U.S. and will very likely remain the most prevalent synthetic opioid threat in the near term' [8]. In this context, it is important to understand that the DEA is referring here to IMF, including analogs, and not prescription fentanyl (such as the transdermal fentanyl patch). See Tables 1 and 2. The DEA reports that there is evidence that transnational criminal organizations (TCOs) operating near the US Southern border are trafficking large quantities of fentanyl mixed with other non-opioid drugs [8].

**Table 1:** Drug nomenclature in the United States

Term	Definition	Comments
Analog	A compound with a chemical structure similar to another compound but with a difference in a certain component; drugs that are structurally similar (but not identical) to another drug	Fentanyl has dozens of analogs and these analogs may not necessarily be 'illegal' as they are new entities
Counterfeit opioid	An illicitly manufactured oral opioid pill created to look like a prescription opioid. Counterfeit oral opioids may even have brand marks like the prescription opioid	Counterfeit opioids are made using pill presses (widely available online), filler material, and some agent to create psychoactive effects, such as IMF
Fentanyl	Fentanyl is a synthetic opioid that exists both as a prescription opioid and as an illicit opioid	Prescription fentanyl such as the transdermal patch is not the source of fentanyl mixed into the illicit drug supply
Heroin	Although originally developed in a legal laboratory around the turn of the century, heroin is illegal in the United States today	Heroin is a Schedule I controlled substance in the United States although some countries use a prescription heroin product for analgesia
Illicit	This refers to activities and processes outside the scope of what is legally sanctioned and controlled	
Illicitly manufactured fentanyl (IMF)	Fentanyl made illegally in clandestine laboratories outside of the law	Not subject to manufacturing standards
Pharmaceutical	A medicinal drug or any drug developed and produced to strict standards and available as a prescription product	
Prescription opioids	Opioid agents manufactured legally under FDA supervision and which may be lawfully prescribed by a physician or other with prescribing privileges	Prescription drugs may be diverted for street sale. Prescription fentanyl is often used in the surgical setting and in a transdermal patch for control of pain in cancer patients.
Synthetic opioids	Opioid agents made entirely in a laboratory setting. Synthetic opioids may be prescription products (such as prescription fentanyl) or illicitly manufactured products	These differ from natural opioids (such as morphine which comes from a plant) and semi-synthetics (such as oxycodone)

**Table 2:** Prescription and illicit fentanyl products. While prescription fentanyl may be diverted for street use (IV fentanyl can be injected, oral products can be taken by mouth), the DEA maintains that this use is small compared to other prescription products. Most fentanyl abuse involves IMF rather than diverted prescription product [9]. IMF: Illicitly manufactured fentanyl

Product	Definition	Comments
Fentanyl buccal tablets	Prescription fentanyl as a tablet that can be dissolved in the mouth	Prescribed for treatment of breakthrough pain in cancer patients
Fentanyl lozenge	Prescription fentanyl in a lozenge or 'lollipop' form	
Illicitly manufactured fentanyl (IMF)	Fentanyl and fentanyl analogs produced illegally in clandestine laboratories, mainly from China. IMF is produced as a powder. It is smuggled into the US by a variety of means and can also be purchased on the dark web using cryptocurrency	These products are 100% diverted to the street drug trade; there is no lawful medical use
Intravenous (IV) fentanyl	Pharmaceutical fentanyl for IV use, typically associated with surgery	These two prescription products account for 98.3% of all fentanyl prescriptions in the US (roughly 3.8 million prescriptions per year)
Transdermal fentanyl	A transdermal or patch system, which releases fentanyl through the skin; this is a prescription pharmaceutical product	

**Figure 3:**
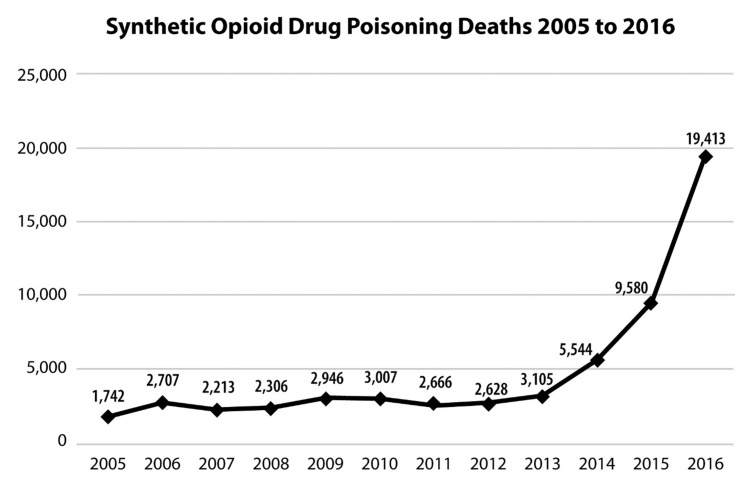
Deaths from synthetic opioid overdose remained relatively stable from 2005 to 2013 but increased sharply with the introduction of illicit fentanyl. Source: Centers for Disease Control and Prevention

Beginning with the 'third wave' of overdose mortality, there has been a marked increase in fatalities associated with synthetic opioids starting around 2013, which is largely attributable to the introduction of IMF into the US illicit drug culture [1,10-12]. See Figure 3. IMF in powder form is primarily marketed to heroin dealers and users, whereas counterfeit pills made with IMF are offered for illicit street sale as 'prescription opioids' [8]. In many cases, the drug user (and in some cases even the drug dealer) are not aware that the product contains IMF and are likewise unaware of the strength, quantity, or purity of the IMF. The mixture of IMF and heroin has become so common on the street that it is occasionally sought out by some illicit drug users who have described the combination as melding the intense psychoactive effects (but short duration of action) of IMF with the milder but more durable psychoactive effects of heroin [8]. It has also been reported that heroin helps buffer the abrupt 'crash' that can occur with IMF alone [8]. While IMF is generally available as a white powder and heroin is somewhat darker in color, it is difficult to distinguish these drugs by sight alone. Drug dealers, users, and law enforcement may all encounter difficulties in telling products apart just from appearance [8].

Transnational criminal organizations (the cartels)

Mexican-based TCOs represent the greatest criminal drug threat to the US and are responsible for trafficking illegal drugs ranging from marijuana to IMF [13]. China and Mexico are the world’s primary manufacturers of IMF and a continuous evolution of fentanyl analogs comes to market to circumvent drug laws, avoid detection, and improve profitability. The main points of entry for IMF into the US occur via international mail or courier services or as substances smuggled over the Southwestern US border with Mexico [13]. In some cases, IMF or its immediate precursors are sent by express, courier, or mail from China to Mexico and then smuggled over the border into the US. While there are many IMF products known to the DEA, brand-new analogs can be developed, which technically may fall outside the scope of the law, and despite their obvious danger may be at least 'not illegal'. Once in the US, IMF in its various forms (IMF, new analogs, precursors) are moved to TCO-controlled stash houses for production and/or subsequent distribution [13]. The challenges to keeping IMF out of the US are manifold: IMF enters through the huge volume of mail and freight into the US; IMF likewise enters over the porous land border in the South; logistical problems in terms of drug detection and inspection can hamper apprehension of IMF; and the murky legal status of some IMF analogs means that even when confiscated, some products may not technically even be illegal.

The profit potential with IMF is a clear motivation for TCOs, who can purchase a kilogram of illicit fentanyl from China for about $5,000 and convert it to products with a market value of upward of $1.5 million [14]. However, the rapid proliferation of cheap, readily available IMF has encouraged participation in this lucrative market by aggressive non-TCO interlopers on the darknet who seek to infringe on the 'territory' of the TCOs. China is a prominent producer of IMF, in particular with respect to precursors, and often ships these items to clandestine labs in Mexico, from which point they enter the US as IMF [14].

There are six major Mexican TCOs involved in IMF trafficking and other crimes. It should be noted that these TCOs have a strong and often violent presence in Mexico, but to a large extent, most of these TCOs keep inter-cartel disputes on Mexican soil [13]. This may change as IMF smuggling increases. See Table 3.

**Table 3:** The nine largest TCOs influencing drug trade on the Southwest border. Source: 2020 Drug Enforcement Administration NDTA [15]. The two best-established cartels, Sinaloa and CJNG, are known to be most associated with trafficking IMF TCO: Transnational criminal organizations

Cartel	Headquarters	Reputation	Main US Areas	Primary Drugs	Comments
Sinaloa	Sinaloa, Mexico	One of the oldest, most established cartels	Pacific Coast, Phoenix, Los Angeles, Denver, Chicago	Methamphetamine, marijuana, cocaine, heroin, IMF	Considered the most active in smuggling IMF over the Southern border
Jalisco New Generation (CJNG)	Guadalajara, Mexico	Very powerful and fastest-growing cartel; reputation for violent altercations with the Mexican government	Los Angeles, New York, Chicago, Atlanta	Cocaine, heroin, methamphetamine, IMF	Active in smuggling along the border and via ports
Juarez (La Linea is a prominent faction)	Chihuahua, Mexico	One of the oldest cartels	El Paso, Denver, Chicago, Oklahoma City	Marijuana, cocaine, heroin, and methamphetamine	Smuggles mainly over Southern border into Southwestern United States
Gulf	Tamaulipas, Mexico	Older TCO with headquarters near US border	Houston, Atlanta, Detroit	Mainly heroin and cocaine	Smuggles mainly into South Texas, especially near Padre Island
Los Zetas (Cartel del Noreste is their most prominent faction)	Nuevo Laredo, Mexico	Splintered off from Gulf Cartel in 2010 and currently involved in the rivalry between two opposing factions (Northeast Cartel and Old School Zetas)	Laredo, Dallas, New Orleans, Atlanta	Cocaine, heroin, methamphetamine, and marijuana	Smuggles mainly into Texas but has diminished somewhat in importance in recent years
Beltran-Leyva Organization	Guerrero, Mexico, and other locations	Split off from Sinaloa in 2008 and operating as small splinter groups.	Phoenix, Los Angeles, Chicago, and Atlanta	Marijuana, cocaine, heroin, and methamphetamine	Has diminished presence in recent years and sometimes partners with other TCOs
Guerreros Unidos	Guerrero, Mexico	Split off from Beltran-Leyva Organization	Los Angeles, New York, Chicago, Atlanta	Primarily heroin	Often partners with Jalisco New Generation
La Familia Michoacána	Michoacán, Mexico	Some affiliations to Jalisco New Generation	Limited	Cocaine, heroin, methamphetamine, IMF	Law enforcement and cartel infighting has decreased prominence of this group
Los Rojos	Reynosa, Mexico	Splinter group of Beltran-Lyeva Organization	Not known	Primarily heroin	Frequent changes in leadership

Drug trafficking over the border also involves money laundering going the other direction, as large shipments of bulk currency must return over the border from the US into Mexico. Los Angeles is a principal hub for drug money laundering back into Mexico because of its strong historical business ties to Mexico, a robust economy, and the presence of numerous large and small banks and financial centers [16]. Cash may also be transported by individuals (hidden in cars or contained in luggage checked on airplanes), sent by mail or courier services, and shipped in by truck. In recent years, Asian-based money laundering has emerged as an important adjunctive service [15,17]. Money laundering by China and other Asian nations has burgeoned in response to growing Asian participation in the drug trade, closer financial ties between Mexican TCOs and Chinese manufacturers of illicit drugs, and the simplified, straightforward procedures for moving bulk currency in and out of certain Asian countries [13]. Seizures of bulk currency in 2017 were highest for California, Arizona, and Ohio ($37, $13, and $2 million, respectively) but it is important to note that this is the amount of cash seized, not the true amount of cash transported [13]. Money laundering can be exceedingly complex, and its constantly evolving methods obscure a cascade of international transactions that can be difficult for forensic accountants to trace [17]. The emergence and proliferation of cryptocurrencies and anonymous financial transactions further complicate the financial side of IMF. On the other hand, money laundering sometimes goes low-tech, in that TCOs sometimes possess sufficient manpower and mobility so that large transactions can be broken down into many small ones and handled by individuals. Another new twist on drug trafficking at the border is the rise of the so-called 'independent contractors', who work on a project basis with no particular organization loyalties; such independent contractors might be engaged to move relatively small amounts of cash or drugs across the border [13]. They typically handle only small amounts of drugs or cash and are often able to operate without earning much attention from law enforcement, who tend to focus on the 'big players'.

The United States Department of Homeland Security (DHS) has briefly but effectively described the complex and highly efficient supply chain for IMF. Raw products and chemical precursors, typically from China, are sent to clandestine labs where they are used to manufacture IMF (including fentanyl analogs). These clandestine labs may be located in China, Mexico, certain Caribbean countries, or even in the US and Canada. It is suspected that sometimes large shipments of street-ready IMF arrive in Mexico from China but are forwarded to their final destinations in the US unopened [18]. The US government has attempted to fight this by designating certain manufacturers and distributors of IMF as consolidated priority organization targets (CPOTs), which exert a high degree of command and control over the most active drug trafficking supply lines and money-laundering systems [19]. However, supply chains and money laundering routes and methods are in a constant state of flux and refinement and have proven to be highly adaptable. Furthermore, the cartels handling this business can be volatile and have to manage sometimes frequent changes in leadership brought on by inter-cartel warfare and law enforcement as well as shifts in supply chain availability and drug use trends [15].

Overall, IMF is an attractive product for drug traffickers, because it can be produced without arousing much attention from the authorities in contrast to heroin or marijuana, which require large amounts of land dedicated to cultivating easily identifiable plants. IMF is marketed mainly in the United States, and foreign countries can readily gain entry into the United States by mail service, courier service, entry points, or being smuggled over the border at or between checkpoints. IMF is sometimes moved by freight forwarders because a chain of freight forwarders can be used to obfuscate the original sender. Many such shipments are sent with deliberately missing shipping details, further making it difficult to track the materials back to the original shipper [20]. Once in the US, the IMF may go to regional distribution points run by criminal enterprises, who may then refine the product with dyes, press them into counterfeit pills, or mix them with inert substances to add to drugs such as heroin. IMF products are then moved to what might be thought of as the 'retail distributors', who either alone or through their own networks, sell the IMF products to the end-users, who may not be aware that they are purchasing IMF. It should be noted that this elaborate train of events is not always necessary, because raw materials, precursors, related equipment, and even IMF itself may be purchased on the darknet using cryptocurrency allowing for anonymity [21]. As such, government officials from the DEA, Customs and Border Patrol, the DHS, the US Postal Inspection Service, Immigration and Customs Enforcement, and state and local law enforcement may all be involved in the apprehension of IMF at various points.

Most of the drugs seized at the Southern border into the US are seized at recognized points of entry, but this may owe to the fact that those points are the most closely monitored crossing points and great expanses of the Southwest border are open, rural, and inadequately patrolled. It is impossible even to estimate the flow of drugs across the open border. Current over-the-border smuggling methods involve taking drugs by hiding them in vehicles (from passenger cars to large trucks), using an elaborate tunnel system that leads from houses controlled by Mexican cartels to safe houses in the US, transporting them in ultralight aircraft, and enlisting pedestrians or others to carry small amounts of illegal drugs with their personal effects. Many of these methods came into play when large quantities of drugs are moved, such as marijuana. IMF smuggling is in many ways easier, as even very small quantities of IMF can be extremely lucrative.

**Figure 4:**
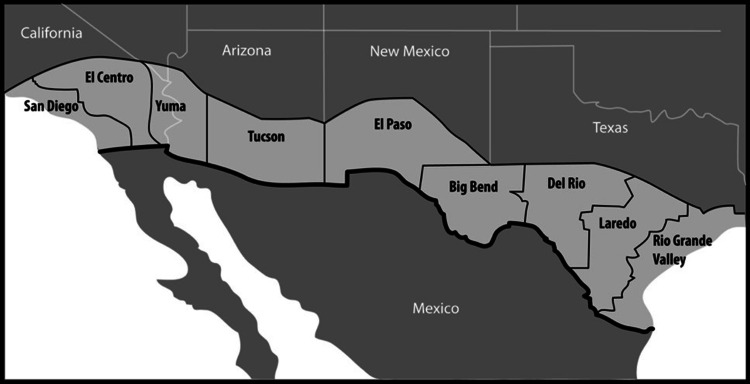
Drug smuggling over the border has been an ongoing concern for decades, but it appears that patterns of drug smuggling are changing. Fentanyl has been reported to date at San Diego and Tucson. Note that marijuana smuggling has gone down in San Diego, El Paso, Big Bend, Del Rio, and Rio Grande Valley; cocaine has decreased substantially in Del Rio. Otherwise, most areas report increases in drug activity [13]. It should be noted that these figures represent confiscated drugs and are likely vastly under-reporting actual drug activity. Map drawn by Todd Cooper, professional artist and owner of Coyote Studios in Green Valley, California, from data provided by the DEA.

About 90% of the heroin in the US enters over the Southwestern border, and the smuggling of IMF appeared to be negligible until February 2019, when the largest quantity of IMF in history was seized at the Southwestern border (over 250 pounds) [22]. See Figure 4.

Seizures of illicit opioids, including but not limited to IMF, have increased markedly in the past few years. IMF entering the US by land tends to be higher volume, lower potency, and about 10% purity compared to the IMF arriving by overseas courier, which is typically smaller in volume but highly potent and may be >90% pure [18]. The explosion of legitimate e-commerce activities in the US has resulted in a consistently high volume of package deliveries, which further obscures fentanyl shipments [18]. Heroin and other opioids are the most frequently smuggled drugs over the border at this time, but IMF appears to be making strong and dangerous inroads. TCOs have been reported to be using their existing infrastructure and organizational systems to incorporate IMF into their product offerings, without necessarily decreasing their trade in other drugs [18].

Individuals who attempt to cross the border from Mexico into the US as migrants may pay a guide (a 'coyote') a fee to assist them in traveling safely over the border. There is a paucity of data on coyote fees, as the only available information comes from interviews or surveys of migrants who may be understandably hesitant to disclose this information. The coyote may work directly for a TCO or an independent contractor who would likely pay a fee to the local TCO for the privilege of staying in business. (TCO members sometimes show up to audit groups to make sure the coyote has paid fees for everybody and is not smuggling anyone 'off the books'.) The Office of Immigration Statistics estimates that only about half of migrants paid a coyote in the 1970s but by 2006, 95% of migrants making the crossing had to pay for these services [23]. Fees for passage are thought to have increased over the years as well and are often paid in installments with a relatively low fee to be paid upfront to get to the staging area and the largest fee due upon reaching the destination. An estimate offered in 2017 suggests that a single immigrant may be asked to pay $1,200 at the staging area and $8,000 or more upon arrival on US soil. In some cases, a relative already in the US may pay the final fee. In interviews with migrants, a few stated that they were offered alternatives to paying such steep fees, such as transporting illegal substances with them over the border or becoming involved in TCO activities in the US after arrival (smuggling, human trafficking) [23]. Such evidence is anecdotal and based on occasional reports.

It has been estimated that 90% of heroin and 90% of illegal cocaine enter the US by being transported over the Southern border with Mexico, but these figures are estimates [12]. It is not clear what percentage of the IMF entering the US comes across the border but reports from US governmental agencies offer statistics to suggest it is increasing rapidly [5,7-9,20]. The role that migrants crossing the border play in drug smuggling (if any at all) is likely negligible. However, activity at the border in certain locations may serve as a diversion to facilitate smuggling by overtaxing border authorities. For instance, migrant activity may be deliberately encouraged by the coyotes in certain areas, while smugglers take advantage of the distraction to move large quantities of drugs over unmonitored areas. It is certainly important to note that the current disorganized border situation parallels an influx of IMF into the country and that failure to better address border security may allow more IMF into the US [24].

The COVID-19 pandemic appears to have had only a minor impact on the trafficking of IMF and other drugs, although it is believed that several cartels withheld regular shipments of methamphetamines into the US to allow for price increases [15]. In the earliest months of the pandemic, there were reportedly problems for cartels in obtaining precursor chemicals for IMF but that problem was transitory and had no sustained impact on IMF production or smuggling [16]. The pandemic did initially affect the transfer of bulk currency via illicit financial networks, in particular, the operations of Chinese-based money laundering operations [15]. Such networks used front companies to transfer funds between China and Hong Kong as well as using underground banking services and mirroring schemes. Many of these networks have counterpart organizations based in the major cities of the US to help facilitate this cash-intensive business. The DEA has reported seeing a recent increase in activity between Chinese money laundering organizations and Mexican cartels [15].

## Conclusions

IMF is driving the current dramatic increase in opioid overdose mortality and there is evidence that some (and likely, in the future, increasing amounts) of this IMF is entering the US over the Southern land border. It is estimated that about 90% of all heroin in the US entered the country from Mexico over the Southern land border and it seems apparent that IMF will follow suit. Since even very small amounts of IMF can be highly profitable, IMF poses new challenges particularly during this time of stress at the Southwest border.
